# Gray and white matter structural examination for diagnosis of major depressive disorder and subthreshold depression in adolescents and young adults: a preliminary radiomics analysis

**DOI:** 10.1186/s12880-022-00892-5

**Published:** 2022-09-12

**Authors:** Huan Ma, Dafu Zhang, Dewei Sun, Hongbo Wang, Jianzhong Yang

**Affiliations:** 1 Department of Radiology, The Third Affiliated Hospital of Kunming Medical University, Kunming, 650018 China; 2 Department of Psychiatry, The Second Affiliated Hospital of Kunming Medical University, 374# DianMian Road, 650101 Kunming, China; 3 Department of Psychiatry, The First Affiliated Hospital of Kunming Medical University, 650018 Kunming, China

**Keywords:** Major depressive disorder, Subthreshold depression, Magnetic resonance imaging, Radiomics, Machine learning

## Abstract

**Background:**

Radiomics is an emerging image analysis framework that provides more details than conventional methods. In present study, we aimed to identify structural radiomics features of gray matter (GM) and white matter (WM), and to develop and validate the classification model for major depressive disorder (MDD) and subthreshold depression (StD) diagnosis using radiomics analysis.

**Methods:**

A consecutive cohort of 142 adolescents and young adults, including 43 cases with MDD, 49 cases with StD and 50 healthy controls (HC), were recruited and underwent the three-dimensional T1 weighted imaging (3D-T_1_WI) and diffusion tensor imaging (DTI). We extracted radiomics features representing the shape and diffusion properties of GM and WM from all participants. Then, an all-relevant feature selection process embedded in a 10-fold cross-validation framework was used to identify features with significant power for discrimination. Random forest classifiers (RFC) were established and evaluated successively using identified features.

**Results:**

The results showed that a total of 3030 features were extracted after preprocessing, including 2262 shape-related features from each T1-weighted image representing GM morphometry and 768 features from each DTI representing the diffusion properties of WM. 25 features were selected ultimately, including ten features for MDD versus HC, eight features for StD versus HC, and seven features for MDD versus StD. The accuracies and area under curve (AUC) the RFC achieved were 86.75%, 0.93 for distinguishing MDD from HC with significant radiomics features located in the left medial orbitofrontal cortex, right superior and middle temporal regions, right anterior cingulate, left cuneus and hippocampus, 70.51%, 0.69 for discriminating StD from HC within left cuneus, medial orbitofrontal cortex, cerebellar vermis, hippocampus, anterior cingulate and amygdala, right superior and middle temporal regions, and 59.15%, 0.66 for differentiating MDD from StD within left medial orbitofrontal cortex, middle temporal and cuneus, right superior frontal, superior temporal regions and hippocampus, anterior cingulate, respectively.

**Conclusion:**

These findings provide preliminary evidence that radiomics features of brain structure are valid for discriminating MDD and StD subjects from healthy controls. The MRI-based radiomics approach, with further improvement and validation, might be a potential facilitating method to clinical diagnosis of MDD or StD.

## Background

Major depressive disorder (MDD) is a category of prevalent, costly, and recurrent mental disease which is one of the leading causes of disability and sub-health worldwide by the global burden of disease study [1]. At present, more than 264 million people are suffering from MDD globally each year [1], and those individuals who only have some of symptoms of depression but do not meet the diagnostic criteria for MDD in terms of the entity, quantity, and duration of symptoms, are known as subthreshold depression (StD) [2], the number of which is immeasurable. Similarly, people with StD show varying degrees of decrement in the health and quality of life [3, 4]. Studies have shown that adolescents and young adults had a higher prevalence rate of StD after age 12 and were at elevated risk for progressing into MDD, and should be given the same concern as patients with MDD [5, 6]. Early detection of depression tendency is helpful to take intervention timely to avoid aggravation of depression [2]. Psychological treatment of StD can prevent the progression to a full-blown MDD [5]. Proactive intervention of StD, also called early treatment or indicated prevention, has been shown to be better effective and cost-effective compared with MDD [5, 6]. Therefore, timely, and active identification of StD from MDD and HC is urgent. The current diagnosis of MDD and StD largely depends on clinical evaluation. Although psychiatrists can relatively easily determine the existence of these two diseases by the subjective experience and clinical symptoms of patients, the diagnostic modality lacks objective markers and may tend to constrain precision in detecting the above two diseases [2, 6].

A variety of neuroimaging techniques, especially MRI, have been widely used in the diagnosis, treatment, and progression monitoring of MDD [7–11]. Previous studies about structural magnetic resonance imaging (sMRI) have extensively reported widespread abnormalities of gray and white matter in MDD patients compared with healthy control subjects, such as alterations in cortical thickness [7, 8], gray and white matter volume [7], surface area [7, 8], and structural connection [9, 10] based on region of interest (ROI), voxel-based morphology (VBM), surface-based morphological analysis (SBA), etc. sMRI studies revealed that the structural dysfunctions exist in multiple brain regions of patients with MDD, mainly including the prefrontal cortex [7, 8, 12], anterior cingulate cortex [7, 12, 13], thalamus [7, 12], amygdala [13, 14], and hippocampus [14]. Previous neuroimaging studies have also showed cerebral anatomical changes in the temporal gyrus and orbitofrontal cortex in people with StD [15–17]. However, it seems that the results are inconsistent and variable, and the brain structural relationship between MDD and StD remains unclear in adolescents and young adults. Up to date, previous studies discriminating MDD from healthy control (HC) or other mental disorders achieved classification accuracy of only about 45.0–85.0% using different modal of imaging and machine-learning methods [12–17]. Many of these methods have not been integrated into a clinical application. We believe the main reason is the heterogeneity of imaging data including data collection, scanning parameters, and processing methods which hampers generalization to other datasets. This makes it difficult to draw comparisons based on the results. In addition, the previous data analyses mostly used conventional group-level statistical methods and such a correlation study did not provide individual-level diagnosis and prediction. Meanwhile, there was few research on the classification of MDD and StD. As such, the exploration of objectively discrimination between MDD and StD and neuroanatomical marker is of great significance for both diagnostic modality and treatment decisions.

In the recent year, the emergence of radiomics has broadened the scope of routine medical imaging, particularly in clinical oncology to extract imaging features in solid tumors [18]. Radiomics is a medical image analysis framework and combines computer technology with single or multiple medical imaging data such as computed tomography (CT), sMRI or functional magnetic resonance imaging (fMRI), positron emission tomography (PET) and single-photon emission computed tomography (SPECT) [18]. The first step of a radiomics workflow for oncology has been to segment tumors on medical images, after which quantitative imaging features are extracted. Imaging features represent the intensity distribution, shape, and texture of tumors and capture distinct phenotypes of tumors that are clinically important [18–20]. Through a series of characterization algorithms, medical images which contain a great deal of valuable information are converted into mineable data, and the macroscopic tissue heterogeneity that cannot be identified by human eyes is quantitatively revealed [18, 19]. Similarly, brain MR images also can be analyzed within a radiomics framework by segmenting anatomic structures and extracting quantitative features to form the mineable dataset. At present, radiomics has been successfully employed to develop imaging biomarkers for neuropsychiatric diseases, such as bipolar disorder (BD) [20], Alzheimer’ s disease (AD) [21], autism spectrum disorder (ASD) [22] and attention deficit hyperactivity disorder (ADHD) [23]. Through extracting and identifying the high-weighted radiomics features, the performance of diagnostic models based on selected radiomics features is excellent than that based on traditional image features, and the highest-weighted features could be used as potential markers [18, 19]. In addition, several studies have demonstrated that the combination of multimodality radiomics features of sMRI was able to improve the discrimination accuracy of diagnosis [18, 19, 24].

To the best of our knowledge, up to date there are few reports on MDD or StD based on radiomics. Moreover, few neuroimaging studies have focused on examining diagnosis and relationship between MDD and StD. Therefore, in this study, we obtained sMRI data from unmedicated adolescents and young adults with MDD, StD and well-matched healthy control subjects. Then, we aimed to identify radiomics features from gray and white matter of each brain region, and establish and validate a radiomics classification model which may address the heterogeneity of imaging studies and facilitate the diagnosis of patients with MDD and StD from healthy controls.

## Methods

### Subjects

This prospective study protocol was approved by the institutional review board of the Third Affiliated Hospital of Kunming Medical University (Kunming, China) according to the principles of the Declaration of Helsinki with the ethical approved number KY2019044. After being thoroughly informed about the purpose and process, the written consent was received from participants ≥ 18 years or assent and permission from parents/guardian for subjects under 18 years old before enrollment. All adolescents and young adult subjects with MDD and StD were right-handed, aged between 13 and 24, and consecutively recruited from September 2019 to December 2020 in the Department of Psychiatry of the First and the Second Affiliated Hospital of Kunming Medical University.

All participants administered a review of medical history records and a complete neuropsychological assessment. Diagnosis of MDD and StD were determined by two psychiatrists (nonauthors, with 5 and 8 years of experience in clinical psychiatry, respectively).

For patients who met the diagnostic criteria of MDD by the Chinese version of the Structured Clinical Interview for Diagnostic and Statistical Manual of Mental Disorders Axis I Disorders, Fifth Edition (DSM-V) or SCID, only patients with unipolar, first-episode and drug-naive depression, then he or she was evaluated directly by 17-items HAMD score. Exclusion criteria included the presence of ① any other current psychiatric disorder, including bipolar disorder, schizophrenia, obsessive compulsive disorder, post-traumatic stress disorder, schizoaffective disorder, depressive disorder associated with substance use or medical conditions; ② history of severe head injury or neurological diseases; ③ suffering from significant or chronic physical illnesses and receiving systematic treatment; ④ history of alcohol and substance abuse or dependence; ⑤ contraindications to MRI examination.

For participants of StD or HC who was recruited from outpatients and from local school via advertisement, he or she was evaluated firstly by using CES-D, then HAMD-17 to rule out MDD. StD patients inclusion criteria were ① Center for Epidemiological Studies Depression Scale (CES-D) score ≥ 16 [25]; ② the scores of 17-items of Hamilton Depression Rating Scale (HAMD_17_) were 7 ~ 17 [26]; ③ not met the diagnostic criteria of MDD and have no history of MDD. The exclusion criteria were the same as those of MDD subjects. Healthy controls (HC) participants were recruited from local school via advertisement. HC were all right-handed and matched with patients for sex and age. The CES-D scores of HC were less than 16, and all HC were reviewed to exclude the presence of any history of psychiatric diseases in first-degree relatives and/or current or previous severe physical or neuropsychiatric illness. There was no any biological relationship between HC and MDD or StD patients. All patients with MDD or StD and HC subjects underwent MRI scan within one week after clinical and psychological evaluation. In total, 147 subjects, including 46 MDD, 50 StD and 51 HCs, were recruited.

### MRI data acquisition

All T1-weighted imaging and DTI data were obtained on a 3.0 Tesla MRI scanner (SIGNA Pioneer, GE Healthcare, Waukesha, WI, USA) equipped with a 21-channel phased-array head coil in the Third Affiliated Hospital of Kunming Medical University (Kunming, China). 3D-BRAVO (BRAin VOlume) was a volumetric magnetization-prepared rapid acquisition gradient echo T1-weighted sequence and was used to obtain high-resolution three-dimensional T1 weighted imaging (3D-T_1_WI) with the following parameters: repetition time (TR) = 8.6ms, echo time (TE) = 3.3 ms, field of view (FOV) = 240 mm× 240 mm, matrix = 256 × 256, number of slices = 340, slice thickness = 1 mm, spacing = 0 mm, flip angle = 12°, Auto-calibrating Reconstruction for Cartesian sampling (ARC) factors = 2, voxel size: 0.9 mm× 0.9 mm× 1 mm, number of slices: 340. Total acquisition time of 3D-T_1_WI was 4 min 29 s. DTI were performed using a single-shot spin-echo echo-planar imaging (SE-EPI) sequence in 64 directions with parameters: TR = 7395 ms, TE = 101.4 ms, FOV = 240 mm × 240 mm, matrix = 130 × 128, slice thickness = 3 mm, spacing = 0 mm, voxel size: 1.9 mm × 1.8 mm × 3.0 mm, Array Spatial Sensitivity Encoding Technique (ASSET) factors = 2, *b*-values = 0 s/mm^2^ and 1000 s/mm^2^. During imaging, foam pads were used to fix the head and participants were instructed to remain stationary. Rubber earplugs were used to abate noise. A neuroradiologist viewed images immediately, and those with obvious artifacts were subjected to rescan. In the present study, data of 5 subjects (3 MDD patients, 1 StD and 1 HC) were excluded because of excessive motion artifacts, and the rest of data from 142 subjects (43 MDD, 49 StD and 50 HCs) were used for preprocessing.

### Data preprocessing and feature extraction

3D-T_1_WI was preprocessed by using the recon-all pipeline of Freesurfer software suite (https://surfer.nmr.mgh.harvard.edu/) as previously described [27]. The first step applied to the T_1_WI was that the DICOM images of each subject were converted to NIFTI format. The auto-recon processing stages contained a series of steps including motion correction and conformation, intensity normalization, skull stripping, inflating and smoothing, spherical mapping and registration, cortical surface reconstruction, cortical parcellation and thickness estimation [23, 27]. The quality of parcellation was visually inspected by overlaying the labeled image on the T_1_WI to refrain from apparent calculation error and invalid topology. Subsequently, the Desikan-Killiany-Tourville (DKT) atlas [27] was used to generate labeled brain, which was divided into 124 labels including 38 cortical and subcortical regions and 24 sulci per hemisphere. DTI data, including eddy-current correction, head movement adjustment, brain segmentation and tensor model fitting, were preprocessed by using FMRIB Software Library (FSL, http://www.fmrib.ox.ac.uk/fsl/) software [28]. DTI of all participants were used to create a study-specific template implemented with Diffusion Tensor Imaging ToolKit (DTI-TK, http://dti-tk.sourceforge.net/). The fractional anisotropy map was generated by registering the template to the Johns Hopkins University of Medicine International Consortium of Brain Mapping diffusion-tensor imaging (JHU-ICBM-DTI) white matter atlas [29] and 48 white-matter regions were labeled. Quality control was performed by visual inspection of the direction-encoding color fractional anisotropic image.

The labeled brains were input into an open-source software called Mindboggle (https://mindboggle.info/) [30]. The features were extracted automatically, including following measures: ① the gray matter morphometry (cortical thickness, mean curvature, convexity, geodesic depth and travel depth) and its statistical metrics i.e., distribution metrics (mean, standard deviation, skew, and kurtosis) of each labeled regions and sulci were extracted from each T_1_WI; ② the diffusion properties of white matter (fractional anisotropy, axial diffusivity, radial diffusivity, mean diffusivity) and its statistical metrics (mean, standard deviation, skew, and kurtosis) of each white matter label were extracted from each DTI.

### Feature selection

The number of the morphometric and diffusion features extracted was enormous. All features were arranged into a feature vector representing both gray and white matter properties of each individual brain. To remove redundant and nonrelevant features that may affect the classification accuracy of the prediction model, we carried out an all-relevant feature selection based on random forest algorithm embedded in a k-fold (k = 10) cross-validation framework by using “Boruta” package in the R software (https://www.r-project.org/) [31] on extracted features. Firstly, the algorithm expanded the given dataset by appending unordered copies of all features, also called shadow features. Secondly, it trained the model, i.e., Random Forest classifier (RFC) on the expanded dataset and estimated the degree of correlation of real features by comparing the importance measure provided by random forest between real features and shadow features. At last, the algorithm examined whether a real feature has a higher degree of correlation to classification than its shadow features at each iteration. The result of the algorithm was to define whether each feature was relevant or irrelevant. In current study, all relevant features were selected and compared among the three groups (MDD, StD and HC), and nonrelevant features that were deemed irrelevant to classification were identified and constantly removed. When the correlation of all features was established, the algorithm was set to terminate.

### Random Forest Classifier training and cross-validation

**Fig. 1:**
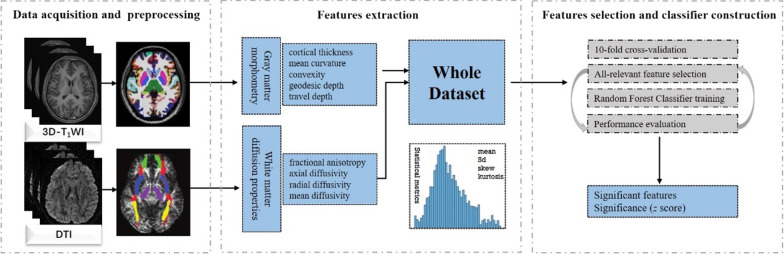
Flowchart of the radiomics process. First, the 3D-T_1_WI and DTI images were acquired and preprocessed. Then radiomics features of shape properties of gray matter, diffusion properties of white matter and their distribution metrics i.e., statistical metrics for each labeled region were extracted. Shape properties included local cortical thickness, mean curvature, convexity, geodesic depth, and travel depth. Diffusion properties contained fractional anisotropy (FA), mean diffusivity (MD), axial diffusivity (AD), and radial diffusivity (RD). Distribution metrics consisted of mean, standard deviation (SD), skew, and kurtosis. In the end, flowchart shows features selection and classifier construction. The all-relevant features selection step was embedded in 10-fold cross-validation. Random forest classifiers (RFC) were established and evaluated to discriminate patients with MDD or StD from healthy controls. Abbreviation: MDD, major depressive disorder; StD, subthreshold depression

RFC was an ensemble algorithm based on decision trees which had superior performance on high-dimension low-sample size problems and required little feature processing and parameter adjustment. RFC for discriminating between MDD and HC subjects, the classifier for discriminating between MDD and StD subjects, and the classifier for discriminating between StD and HC subjects were constructed and evaluated through the workflow shown in Fig. 1. The feature selection was embedded in a repeated k-fold (k = 10) cross-validation framework [31] to achieve unbiased assessments of the true classification error with the R package caret. In each cross-validation loop, the whole dataset of the features was randomly divided into ten nonoverlapping subsets in equal size. Nine subsets were fed into the all-relevant feature selection step and used to train the RFC as the training set, and one remaining subset of selected features was used as the testing set to evaluate the performance of the model. In order to obtain more accurate results and stable performance estimation, we conducted 100 runs of 10-fold cross-validation and summarized the classifier performance from a total of 1000 training-testing cycles [31, 32]. The mean value of the results of 10 runs was taken as the performance of the classifier. Finally, the area under curve (AUC), accuracy, sensitivity, and specificity were used to evaluate the performance of the classifier.

### Statistical analysis of the relevance of selected features

In our workflow, features were selected in each iteration on different subsets of features taken from the cross-validation procedure. Features that were selected in more iterations than would be expected to occur at random were identified as significantly relevant feature [31, 32]. To define the relevance of selected features, 1000 random datasets were created by permuting the original dataset. The expected distribution of selection frequency, i.e., z score of each feature throughout cross-validation iterations was modeled as a binomial distribution with the parameter estimated as the mean selection frequency in all random data sets. This distribution was then used to find features in the original dataset with selection frequency significantly higher than would be expected by chance, with adjusted *P* values of 0.05 [31, 32].

## Results

### Demographic comparison

In Table 1, we listed the demographic variables and clinical information for all participants in current study. No significant difference was detected with respect to the gender, age, education among MDD, StD and HC, while significant difference was observed for CES-D score between StD and HC, and for HAMD17 score among these three groups. HAMD_17_ score was significantly different (*P* < 0.001), with MDD patients having the highest scores and HC subjects having the lowest scores.

**Table 1:** Demographic and neuropsychological data for MDD, StD and HC subjects

Characteristics	MDD (n = 43)	StD (n = 49)	HC (n = 50)	Statistics	*P* Value
Age(years)	16.3 ± 2.3	17.2 ± 3.4	16.9 ± 2.7	0.430	0.674
Gender(M/F)	12/31	15/34	16/34	1.178*	0.278
Education(years)	9.9 ± 2.1	10.1 ± 2.7	9.5 ± 2.8	0.689	0.550
CES-D score	NA	22.7 ± 1.3	11.4 ± 1.6	75.68	0.000
HAMD_17_ score	23.4 ± 2.5	11.6 ± 2.1	3.3 ± 2.2	173.2	0.000

Note: Unless otherwise indicated, data are means ± standard deviation; Unless otherwise indicated, statistics were calculated with ANOVA; * Chi-square test (χ2) test was used

Abbreviation: MDD, major depressive disorder; StD, subthreshold depression; HC, healthy control; CES-D, Center for Epidemiological Studies Depression Scale; HAMD_17_, 17-items of Hamilton Depression Rating Scale; M, Male; F, Female; NA, Not Applicable

### Feature selection and classification performance

**Fig. 2:**
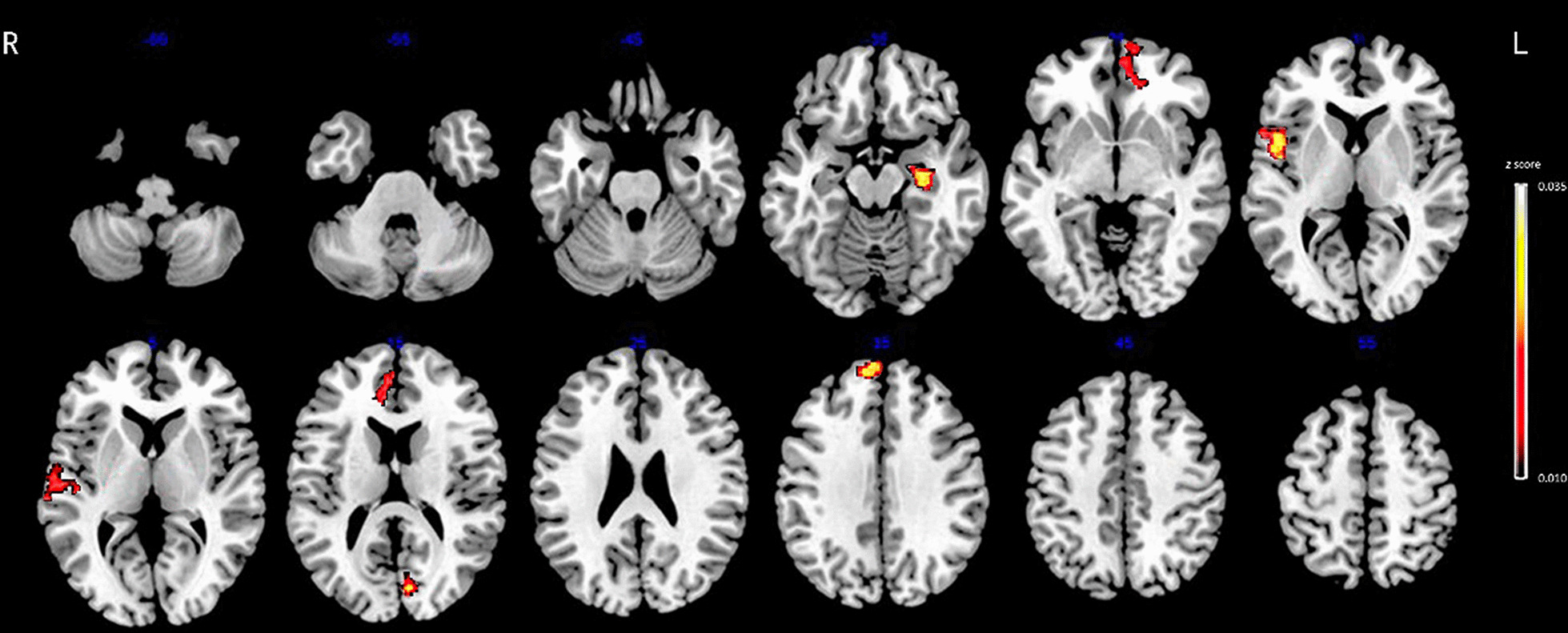
Ten radiomics features of sMRI for discriminating MDD and HC with significant radiomics features located in the left medial orbitofrontal, right superior and middle temporal regions, right anterior cingulate, left cuneus and hippocampus. Abbreviation: MDD, major depressive disorder; HC, healthy control

**Fig. 3:**
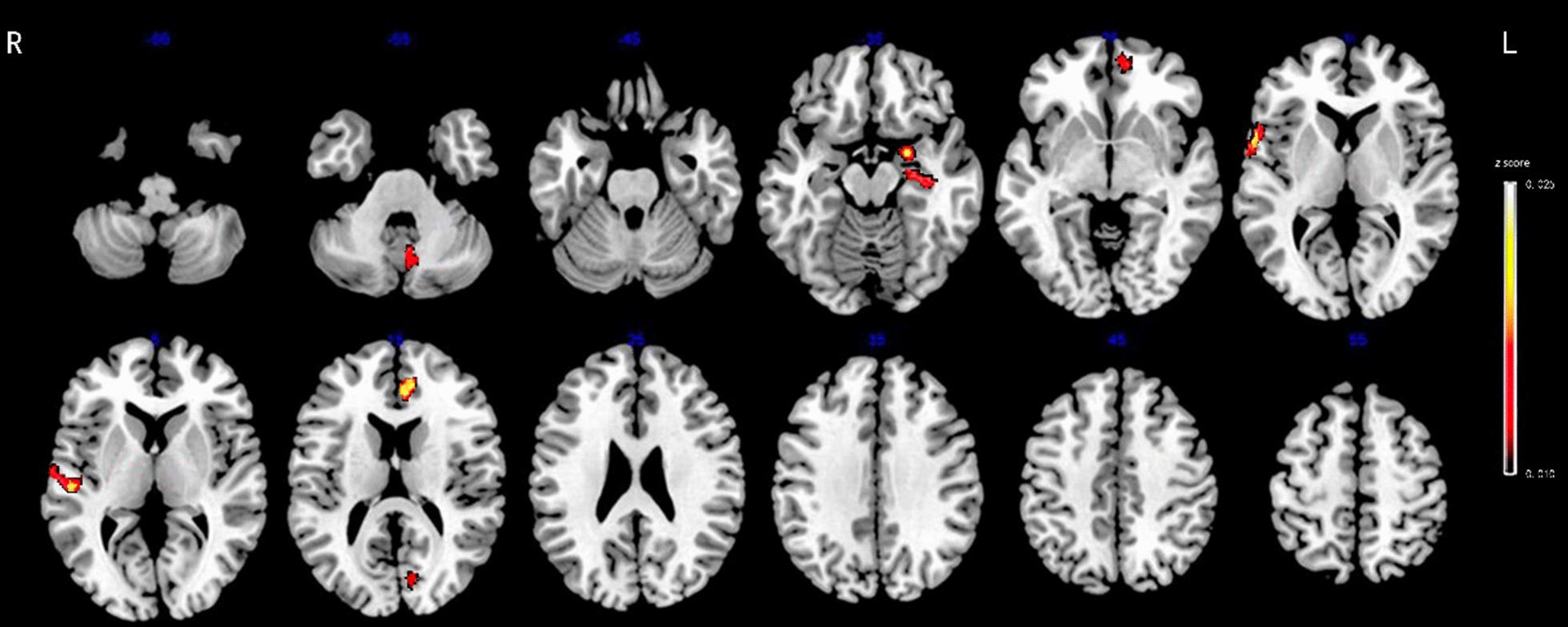
Eight radiomics features of sMRI for discriminating StD and HC with significant radiomics features located in the left cuneus, medial orbitofrontal, cerebellar vermis, hippocampus, anterior cingulate and amygdala, right superior and middle temporal regions. Abbreviation: StD, subthreshold depression; HC, healthy control

**Fig. 4:**
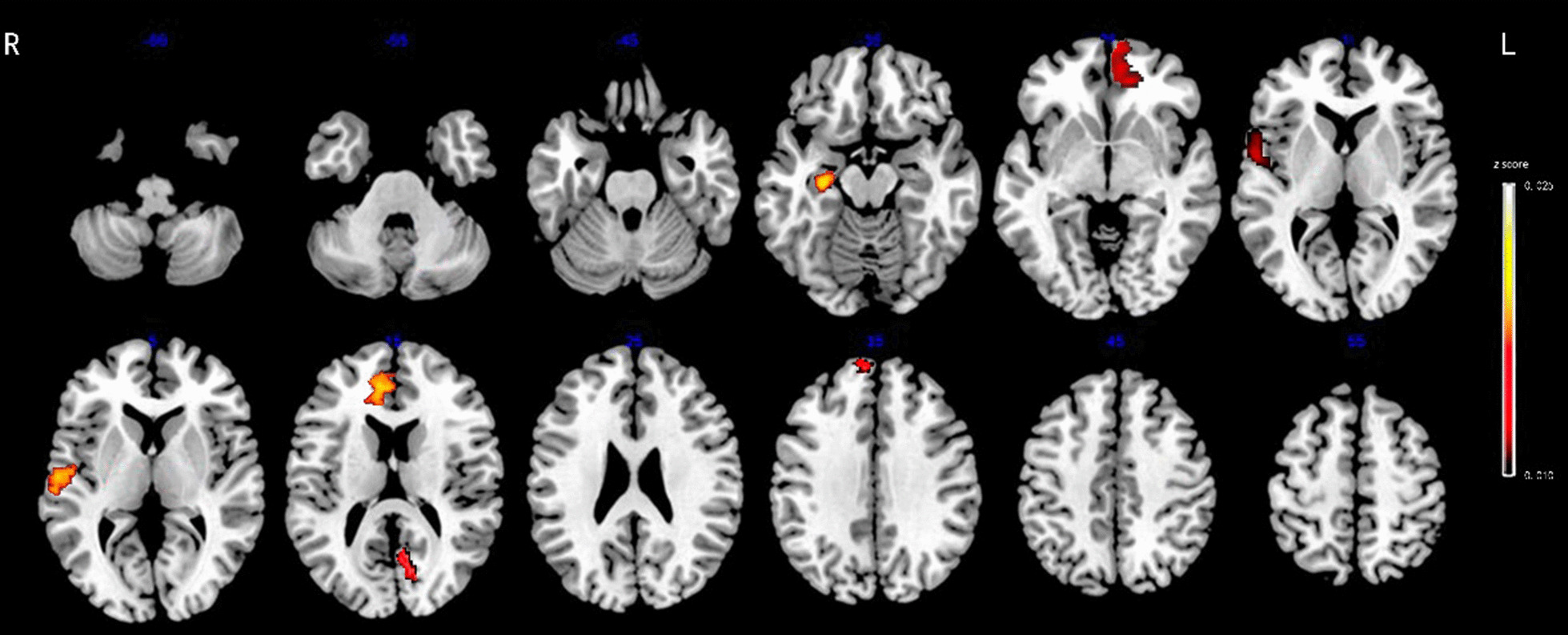
Seven radiomics features of sMRI for discriminating MDD and StD with significant radiomics features located in the left medial orbitofrontal, middle temporal, cuneus, and right superior frontal, superior temporal regions and hippocampus, anterior cingulate. Abbreviation: MDD, major depressive disorder; StD, subthreshold depression

A total of 3030 features were extracted from gray and white matter structural examination, including 2262 shape-related features from each T1-weighted image representing gray-matter morphometry and 768 features from each DTI representing the diffusion properties of white-matter. During building the random forest classifier to discriminate between MDD, StD from HC subjects, the mean number of features in each subset was 11.3 (range, seven to fifteen), only 0.23%~0.49% of all features, and seven to ten features were identified as significantly relevant because of higher significance, i.e., *z* score. Tables 2, 3 and 4; Figs. 2, 3 and 4 show the relative significance of features extracted from the brain regions.

**Table 2:** Significant features for differentiating MDD patients from HC subjects

Hemisphere	Brain region	Type of features	Detailed features	Significance (*z* score)
R	Middle temporal	Convexity	Kurtosis	0.0340
R	Superior frontal	Mean curvature	Skew	0.0318
L	Cuneus	Local thickness	Mean	0.0253
L	Hippocampus	Geodesic depth	Mean	0.0222
L	Medial orbitofrontal	Local thickness	Mean	0.0202
R	Superior temporal	Mean curvature	Kurtosis	0.0137
R	Anterior cingulate	Mean curvature	Kurtosis	0.0119
L	Medial orbitofrontal	Travel depth	Skew	0.0115
R	Anterior cingulate	Local thickness	Mean	0.0102
L	Medial orbitofrontal	Convexity	Skew	0.0092

R, right; L, left. Type of features: cortical thickness, mean curvature, convexity, geodesic depth, travel depth, fractional anisotropy, axial diffusivity, radial and mean diffusivity. Detailed features: mean, standard deviation, skew and kurtosis

**Table 3:** Significant features for differentiating StD patients from HC subjects

Hemisphere	Brain region	Type of features	Detailed features	Significance (*z* score)
R	Superior temporal	Convexity	Kurtosis	0.0255
R	Middle temporal	Local thickness	Skew	0.0227
L	Anterior cingulate	Local thickness	Kurtosis	0.0221
L	Amygdala	Mean curvature	Kurtosis	0.0185
L	Cuneus	Local thickness	Mean	0.0131
L	Medial orbitofrontal	Mean curvature	Skew	0.0121
L	Cerebellar vermis	Convexity	Mean	0.0091
L	Hippocampus	Mean curvature	Skew	0.0090

R, right; L, left. Type of features: cortical thickness, mean curvature, convexity, geodesic depth, travel depth, fractional anisotropy, axial diffusivity, radial and mean diffusivity. Detailed features: mean, standard deviation, skew and kurtosis

**Table 4:** Significant features for differentiating between MDD and StD subjects

Hemisphere	Brain region	Type of features	Detailed features	Significance (*z* score)
L	Medial orbitofrontal	Local thickness	Mean	0.0253
R	Superior frontal	Mean curvature	Kurtosis	0.0231
R	Superior temporal	Mean curvature	Kurtosis	0.0178
L	Middle temporal	Local thickness	Skew	0.0125
R	Hippocampus	Mean curvature	Skew	0.0112
R	Anterior cingulate	Local thickness	Mean	0.0111
L	Cuneus	Local thickness	Mean	0.0108

R, right; L, left. Type of features: cortical thickness, mean curvature, convexity, geodesic depth, travel depth, fractional anisotropy, axial diffusivity, radial and mean diffusivity. Detailed features: mean, standard deviation, skew and kurtosis

**Fig. 5:**
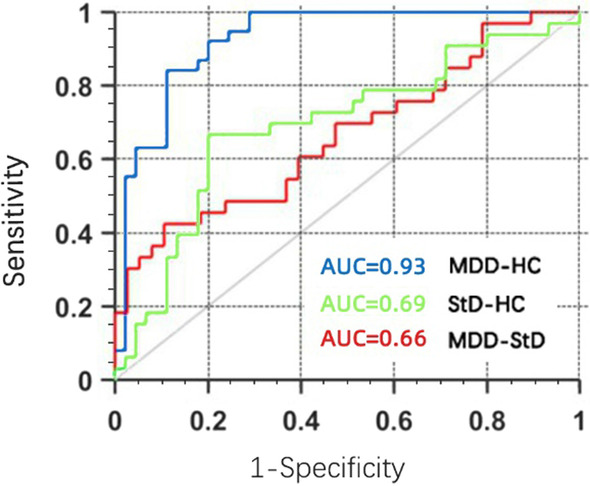
Receiver operating characteristic(ROC)curve of the random forest model for discriminating between MDD and HC subjects (blue line), StD and HC subjects (green line) and MDD and StD subjects (red line). Abbreviation: MDD, major depressive disorder; StD, subthreshold depression; HC, healthy control

After cross-validation, the classifier performance was summarized and the classifiers were evaluated for discriminating MDD, StD from HC subjects. The AUC, accuracy, sensitivity, and specificity of the random forest classifiers were 0.93, 86.75%, 84.21%, 88.89% for discriminating between MDD and HC with significant features located in the left medial orbitofrontal cortex, right superior and middle temporal regions, right anterior cingulate, left cuneus and hippocampus, 0.69, 70.51%, 57.58%, 80.00% for distinguishing StD from HC with significant features located in left cuneus, medial orbitofrontal cortex, cerebellar vermis, hippocampus, anterior cingulate and amygdala, right superior and middle temporal regions, 0.66, 59.15%, 54.55%, 63.16% for differentiating MDD from StD with significant features located in left medial orbitofrontal cortex, middle temporal and cuneus, right superior frontal gyrus, superior temporal regions and hippocampus, anterior cingulate, respectively. Table 5; Fig. 5 show the ROC and AUC obtained by the RFC for classifying between MDD, StD and HC subjects.

**Table 5:** Classification performance for random forest classifiers using radiomics features

	MDD - HC	StD - HC	MDD - StD
Accuracy	86.75	70.51	59.15
Sensitivity	84.21	57.58	54.55
Specificity	88.89	80.00	63.16

MDD, major depressive disorder; StD, subthreshold depression; HC, healthy control

## Discussion

Based on radiomics analysis, the major finding of this study indicated that the radiomic-based classifiers could provide moderate diagnostic value by using cerebral sMRI features in discriminating MDD or StD from healthy controls, especially in distinguishing MDD from healthy controls with excellent classification accuracy. The majority of gray matter morphometry alteration that contributed to the discrimination was located within left medial orbitofrontal lobe, right superior frontal gyrus, right superior and middle temporal regions, bilateral anterior cingulate and hippocampus, left cuneus, amygdala, and cerebellar vermis. This is a preliminary study to identify structural radiomics features of gray and white matter using radiomics analysis. Our results are consistent with the previous MRI findings based on traditional data analysis [7, 9, 12–14], and in accordance with the pathological hypothesis of the limbic-cortico-striatal-pallidal-thalamic (LCSPT) circuits [33]. In our study, the AUC and accuracy the classifier achieved were 0.93 and 86.75% respectively in classification of MDD and normal controls, which show the better classification performance than voxel-based morphometry [7, 9, 34–36]. This also suggested that structural changes of medial orbitofrontal cortex, temporal lobe, hippocampus, and anterior cingulate gyrus could be potential imaging features for quantitative diagnosis of MDD patients. Although the classifier had excellent diagnostic performance for discriminating MDD from HC, the effect of classifiers for classifying MDD and StD, StD and HC were not good enough. Although radiomics can find more information than routine analysis of brain imaging by deeply mining and selecting the features that carry useful information related to the changes of brain structure in depression, it is crucial to be deeply concerned that radiomics features may present the high-dimension low-sample size problem [18–20]. Common practice in machine learning is to perform feature selection to reduce the dimensionality by selecting the features that carry useful information related to category label. However, like the issue of feature selection in genetics research, the features from neuroimaging data can also be highly correlated because of the intrinsic network architecture of the human brain [19, 20]. Thus, we identified all the relevant features related to the disease during the classification process. The issue not only highlights the advantages of radiomics, but also illustrates the difficulties and shortcomings of radiomics which required tremendous data for model training [18, 19, 24].

Our results revealed all three brain regions including medial orbitofrontal cortex, superior temporal regions and anterior cingulate were identified to be significantly different among the HC versus StD and StD versus MDD. Previous neuroimaging studies have also showed that patients with MDD and StD had relatively smaller GM volume in the temporal gyrus and orbitofrontal cortex than healthy controls, and the decreasing degree of StD subjects was less than that in the patients with MDD [15–17]. In addition, the orbitofrontal cortex has been implicated in many neuropsychological functions such as emotion regulation, evaluation, reward-based decision-making, impulse control etc., [37, 38]. These finding suggested that pathophysiological trajectory process in these gyri might be involved in the transformation of brain structures from HC to StD and to MDD which seems like a continuous spectrum of what is happening in the brain structure, and depressive disorders should be better treated as a spectrum disorder [17].

There are few studies examining cerebellar anatomical changes in MDD and StD [39, 40]. In this study, we observed that in addition to the brain regions of the LCSPT circuits, the structural radiomics feature of cerebellar vermis also played an important role in distinguishing StD from healthy controls. A previous study found that patients with StD showed cerebellar volume loss, and current subthreshold depressive symptoms were associated with microstructural changes in brain regions known to be involved in MDD [41]. Therefore, our findings were in line with previous study and illustrated that cerebellum played a possible role in the pathophysiology of StD emerging and progression, and might be a potential imaging marker in detection and monitoring of StD.

DTI characterizes the alterations in WM microstructural properties that cannot be measured using conventional anatomical MRI in vivo. Numerous studies demonstrated that MDD patients had lower FA accompanied by higher RD, yet no differences in MD or AD in callosal, association, and commissural fibers [42–45]. Subtle, but widespread abnormalities of WM in MDD patients were found within the corpus callosum, corona radiata, cingulum, internal capsule, fronto-occipital fasciculus, and fornix. Furthermore, it seems that WM microstructural changes were more common in adult MDD patients with an age of onset over 21 years and more than one episode of MDD [44, 45]. However, previous results have been inconsistent in the pattern of deficits, and the degree of disruption across studies [42–44]. Unfortunately, no significant WM radiomics features were found in our study contributed to discriminating MDD and StD from controls. Therefore, we need to increase the sample size for further research in the future.

## Limitations

Our study has several limitations that need to be taken into consideration when interpreting the results. Firstly, considering the high-dimensional imaging data quite often includes a limited number of samples, determining an effective and optimal approach to diagnose MDD is particularly challenging. We need to combine the type of machine learning algorithms and the number of selected features to estimate the sample size. Based on our findings and properties of radiomics, large-scale multicenter datasets and extraction of all the relevant features may improve the performance of the radiomic classifier. In addition, the study of multimodal data combined with clinical information, radiomics and genomics will be more meaningful and represents promising avenues for future research [46, 47]. Secondly, in order to minimize the impact of age-related brain structural changes, the present study focused on adolescents and young adults, however, adolescents and young adults are still at different age span of their neurological developmental trajectories [48]. This heterogeneity of maturity may be an interactive factor which influences the classification performance. Finally, whether patients with MDD or StD who were correctly identified using radiomics features of brain MRI represent a biological subtype with diverse pathophysiological substrate, prognosis or treatment outcome remains to be investigated in future studies [2].

## Conclusion

In general, this paper presented a radiomic approach using structural radiomics features derived from gray and white matter to discriminate MDD and StD individuals from healthy controls in adolescents and young adults. Our preliminary results show that the sMRI-based radiomics analysis, with further improvement and validation, might be a potential facilitating method to clinical diagnosis of MDD or StD.

## Data Availability

The datasets analyzed in this study are available from the corresponding author on request.

## References

[1] GBD 2017 Disease and Injury Incidence and Prevalence Collaborators (2018). Global, regional, and national incidence, prevalence, and years lived with disability for 354 diseases and injuries for 195 countries and territories, 1990–2017: a systematic analysis for the Global Burden of Disease Study 2017. Lancet.

[2] Gilbody S, Lewis H, Adamson J, Atherton K, Bailey D, Birtwistle J (2017). Effect of collaborative care vs usual care on depressive symptoms in older adults with subthreshold depression: the CASPER randomized clinical trial. JAMA.

[3] Chachamovich E, Fleck M, Laidlaw K, Power M (2008). Impact of major depression and subsyndromal symptoms on quality of life and attitudes toward aging in an international sample of older adults. Gerontologist.

[4] Tuithof M, Ten-Have M, Dorsselaer S, Kleinjan M, Beekman A, de Graaf R (2018). Course of subthreshold depression into a depressive disorder and its risk factors. J Affect Disord.

[5] Cuijpers P, Pineda BS, Ng MY, Weisz JR, Muñoz RF, Gentili C (2021). A meta-analytic review: psychological treatment of subthreshold depression in children and adolescents. J Am Acad Child Adolesc Psychiatry.

[6] Cuijpers P, Quero S, Dowrick C, Arroll B (2019). Psychological treatment of depression in primary care: recent developments. Curr Psychiatry Rep..

[7] Foland-Ross LC, Sacchet MD, Prasad G, Gilbert B, Thompson PM, Gotlib IH (2015). Cortical thickness predicts the first onset of major depression in adolescence. Int J Dev Neurosci.

[8] Hilbert K, Lueken U, Muehlhan M, Beesdo-Baum K (2017). Separating generalized anxiety disorder from major depression using clinical, hormonal, and structural MRI data: a multimodal machine learning study. Brain Behav.

[9] Nguyen KP, Fatt CC, Treacher A, Mellema C, Trivedi MH, Montillo A (2019). Predicting response to the antidepressant bupropion using pretreatment fMRI. Predict Intell Medi.

[10] Chang B, Choi Y, Jeon M, Lee J, Han KM, Kim A (2019). ARPNet: antidepressant response prediction network for major depressive disorder. Genes.

[11] Bartlett EA, DeLorenzo C, Sharma P, Yang J, Zhang M, Petkova E (2018). Pretreatment and early-treatment cortical thickness is associated with SSRI treatment response in major depressive disorder. Neuropsychopharmacology.

[12] Qiu L, Lui S, Kuang W, Huang X, Li J, Li JX (2014). Regional increases of cortical thickness in untreated, first-episode major depressive disorder. Transl Psychiatry.

[13] Van Eijndhoven P, Mulders P, Kwekkeboom L, van Oostrom I, van Beek M, Janzing J (2016). Bilateral ECT induces bilateral increases in regional cortical thickness. Transl Psychiatry.

[14] Zorlu N, Cropley VL, Zorlu PK, Delibas DH, Adibelli ZH, Baskin EP (2017). Effects of cigarette smoking on cortical thickness in major depressive disorder. J Psychiatr Res.

[15] Webb CA, Weber M, Mundy EA, Killgore WD (2014). Reduced gray matter volume in the anterior cingulate, orbitofrontal cortex and thalamus as a function of mild depressive symptoms: a voxel-based morphometric analysis. Psychol Med.

[16] Schmaal L, Hibar DP, Sämann PG, Hall GB, Baune BT, Jahanshad N (2017). Cortical abnormalities in adults and adolescents with major depression based on brain scans from 20 cohorts worldwide in the ENIGMA Major Depressive Disorder Working Group. Mol Psychiatry.

[17] Zhang T, Zhao B, Shi C, Nie B, Liu H, Yang X (2020). Subthreshold depression may exist on a spectrum with major depressive disorder: evidence from gray matter volume and morphological brain network. J Affect Disord.

[18] Gillies RJ, Kinahan PE, Hricak H (2016). Radiomics: images are more than pictures, they are data. Radiology.

[19] Avanzo M, Wei L, Stancanello J, Vallières M, Rao A, Morin O (2020). Machine and deep learning methods for radiomics. Med Phys.

[20] Wang Y, Sun K, Liu Z, Chen G, Jia Y, Zhong S (2020). Classification of unmedicated bipolar disorder using whole-brain functional activity and connectivity: a radiomics analysis. Cereb Cortex.

[21] Won SY, Park YW, Park M, Ahn SS, Kim J, Lee SK (2020). Quality reporting of radiomics analysis in mild cognitive impairment and Alzheimer’s Disease: a roadmap for moving forward. Korean J Radiol.

[22] Chaddad A, Desrosiers C, Hassan L, Tanougast C (2017). Hippocampus and amygdala radiomic biomarkers for the study of autism spectrum disorder. BMC Neurosci.

[23] Sun H, Chen Y, Huang Q, Lui S, Huang X, Shi Y (2018). Psychoradiologic utility of MR imaging for diagnosis of attention deficit hyperactivity disorder: a radiomics analysis. Radiology.

[24] Zhou H, Jiang J, Lu J, Wang M, Zhang H, Zuo C (2019). Dual-model radiomic biomarkers predict development of mild cognitive impairment progression to Alzheimer’s Disease. Front Neurosci.

[25] Radloff LS (1977). The CES-D scale: a self-report depression scale for research in the general population. Appl Psychol Meas.

[26] Demyttenaere K, De Fruyt J (2003). Getting what you ask for: on the selectivity of depression rating scales. Psychother Psychosom.

[27] Klein A, Tourville J (2012). 101 labeled brain images and a consistent human cortical labeling protocol. Front Neurosci.

[28] Jenkinson M, Beckmann CF, Behrens TE, Woolrich MW, Smith SM (2012). FSL NeuroImage.

[29] Mori S, Oishi K, Jiang H, Jiang L, Li X, Akhter K (2008). Stereotaxic white matter atlas based on diffusion tensor imaging in an ICBM template. NeuroImage.

[30] Klein A, Ghosh SS, Bao FS, Giard J, Häme Y, Stavsky E (2017). Mindboggling morphometry of human brains. PLoS Comput Biol.

[31] Kursa MB, Rudnicki WR (2010). Feature selection with the Boruta package. J Stat Softw.

[32] Varma S, Simon R (2006). Bias in error estimation when using cross-validation for model selection. BMC Bioinformatics.

[33] Soares JC, Mann JJ (1997). The anatomy of mood disorders: review of structural neuroimaging studies. Biol Psychiatry.

[34] Sankar A, Zhang T, Gaonkar B, Doshi J, Erus G, Costafreda SG (2016). Diagnostic potential of structural neuroimaging for depression from a multi-ethnic community sample. BJPsych open.

[35] Singh MK, Kesler SR, Hadi Hosseini SM, Kelley RG, Amatya D, Hamilton JP (2013). Anomalous gray matter structural networks in major depressive disorder. Biol psychiatry.

[36] Liao Y, Huang X, Wu Q, Yang C, Kuang W, Du M (2013). Is depression a disconnection syndrome? meta-analysis of diffusion tensor imaging studies in patients with MDD. J Psychiatry Neurosci.

[37] Rudebeck PH, Saunders RC, Prescott AT, Chau LS, Murray EA (2013). Prefrontal mechanisms of behavioral flexibility, emotion regulation and value updating. Nat Neurosci.

[38] Murray EA, Rudebeck PH (2018). Specializations for reward-guided decision-making in the primate ventral prefrontal cortex. Nat Rev Neurosci.

[39] Yucel K, Nazarov A, Taylor VH, Macdonald K, Hall GB, Macqueen GM (2013). Cerebellar vermis volume in major depressive disorder. Brain Struct Funct.

[40] Phillips JR, Hewedi DH, Eissa AM, Moustafa AA (2015). The cerebellum and psychiatric disorders. Front Public Health.

[41] Peng J, Liu J, Nie B, Li Y, Shan B, Wang G (2011). Cerebral and cerebellar gray matter reduction in first-episode patients with major depressive disorder: a voxel-based morphometry study. Eur J Radiol.

[42] Ota M, Noda T, Sato N, Hattori K, Hori H, Sasayama D (2015). White matter abnormalities in major depressive disorder with melancholic and atypical features: a diffusion tensor imaging study. Psychiatry Clin Neurosci.

[43] Olvet DM, Delaparte L, Yeh FC, DeLorenzo C, McGrath PJ, Weissman MM (2016). A comprehensive examination of white matter tracts and connectometry in major depressive disorder. Depress Anxiety.

[44] Westlye LT, Walhovd KB, Dale AM, Bjørnerud A, Due-Tønnessen P, Engvig A (2010). Life-span changes of the human brain white matter: diffusion tensor imaging (DTI) and volumetry. Cereb Cortex.

[45] Van Velzen LS, Kelly S, Isaev D, Aleman A, Aftanas LI, Bauer J (2020). White matter disturbances in major depressive disorder: a coordinated analysis across 20 international cohorts in the ENIGMA MDD working group. Mol Psychiatry.

[46] Huang YQ, Liang CH, He L, Tian J, Liang CS, Chen X (2016). Development and validation of a radiomics nomogram for preoperative prediction of lymph node metastasis in colorectal cancer. J Clin Oncol.

[47] Huang K, Lin Y, Yang L, Wang Y, Cai S, Pang L (2020). A multipredictor model to predict the conversion of mild cognitive impairment to Alzheimer’s disease by using a predictive nomogram. Neuropsychopharmacology.

[48] Lee YY, Stockings EA, Harris MG, Doi S, Page IS, Davidson SK (2019). The risk of developing major depression among individuals with subthreshold depression: a systematic review and meta-analysis of longitudinal cohort studies. Psychol Med.

